# Treatment of Epistaxis

**Published:** 1888-01

**Authors:** 


### *TREATMENT OF EPISTAXIS.*

Dr. J. Robinson, of Kansas, speaking of the treatment of this affection, in the *Therapeutic Gazette*, says:

It is a well-known fact to anatomists and others, that the hemorrhage in the vast majority of cases proceeds from the septum-nares, and is supplied by a branch of the superior coronary, a branch of the facial, which ramifies in the septum-nares. It enters the opening of the nose just below the alae nasi, crossing the superior maxillary bone at that point.

Now, in a practice of nearly thirty years, I have had many cases of epistaxis, and have never in a single case failed to arrest the bleeding by compression of the aforesaid artery, with the finger applied over its track, making firm pressure against the bone. This will arrest the bleeding in nine hundred and ninety-nine cases in a thousand. I have been called to see cases when other physicians had plugged the nostrils, and injected solutions of ferri persulphas, ice-water, etc., without benefit, and have at once arrested all hemorrhage instantly by the above simple means. Tell them to try it.

